# Editorial: Advancements in protein modification for enhanced digestibility

**DOI:** 10.3389/fnut.2025.1603394

**Published:** 2025-04-11

**Authors:** Sajad Shokri, Marcela Jarpa-Parra

**Affiliations:** ^1^Department of Chemical Engineering, University of Bath, Bath, United Kingdom; ^2^Natural Resources and Polymers Research Laboratory, Universidad Adventista de Chile, Chillán, Chile

**Keywords:** protein digestibility, plant-based proteins, protein modification, human health, anti-nutritional factors, gut microbiome, nutritional quality

Proteins play an essential role as structural and functional components to maintain growth and other physiological functions in humans. Amino acids, the fundamental building blocks of proteins, are vital for maintaining the function of all organs, brain, hormones, muscles, biological fluids, and immune system. Factors such as amino acid composition of dietary proteins and their digestibility determine the capacity of a food protein to satisfy metabolic demands for amino acids and nitrogen (1).

Protein digestibility determines the quantity of dietary protein that is effectively digested and absorbed by the digestive system, thus making it adequately available for metabolic activities. Protein digestibility can be influenced by several factors, including protein source, protein structure, their amino acid composition, presence of anti-nutritional factors, food processing, food matrix, individual differences, and others (2).

The importance of enhancing protein digestibility is steadily gaining recognition, driven by various factors closely linked to human health and nutrition. Increased protein digestibility, characterized by a higher biological value, is a key factor in optimizing protein utilization, supporting various aspects of health, and promoting a balanced and nutritious diet. On the other hand, there is a growing interest in exploring new protein sources as alternatives to replace animal-based proteins, such as plant-based proteins. Plant proteins are considered healthier and offer a sustainable protein source in response to the rising global demand for protein consumption. However, proteins derived from plant sources may pose challenges to efficient digestion due to complexity of plant matrices and the presence of antinutritional compounds (3). As a result, there is a growing focus on research efforts aimed at enhancing the digestibility of plant-based proteins.

Increased protein digestibility can be achieved through various methods and strategies, depending on the protein source and the specific challenges it presents. Thus, this Research Topic is aimed at collecting articles those investigate how advanced protein modification methods can lead to more easily digestible proteins for enhanced nutritional benefits and improved human health.

Enzymatic hydrolysis has been shown to enhance the functional properties and digestibility of proteins by breaking down complex polypeptides into smaller peptides and free amino acids, thereby improving their solubility, bioavailability, and techno-functional applications in various food and pharmaceutical formulations (4). The first study in the Research Topic used acid-active proteases to optimize dietary protein digestibility of various protein sources (Mak et al.). This study introduces a family of acid-active bacterial proteases (S53) to enhance the digestibility and nutritional quality of various protein sources. Using the INFOGEST 2.0 protocol, the researchers assessed the effectiveness of these proteases on animal and plant-based proteins, such as soy, pea, chickpea, rice, casein, and whey. Results showed that the most effective protease from the S53 family increased protein digestibility by 115% during the gastric phase and 15% during the intestinal phase, based on the degree of hydrolysis. The widespread adoption of these proteases could enhance protein nutritional value, contributing to food security and sustainability while benefiting populations with high protein needs or compromised digestive systems (Mak et al.).

The second work evaluated the role of dual hydrolysis of soybean on functional properties and palatability of soybean (Jogi et al.). This study investigates how modifying soybean hydrolysis can enhance its functional properties and palatability. Soy protein hydrolysate (SPH) was prepared using Alcalase for 4 h as a control, while modified hydrolysis (MPH) was performed by sequentially hydrolyzing the supernatant with 50% of the enzyme at each stage. Results showed that MPH had higher protein content than SPH and non-hydrolyzed soybeans. Additionally, MPH exhibited a greater degree of hydrolysis and improved functional properties. The hardness of retrograded corn starch was significantly reduced in MPH during storage at 4°C. Sensory analysis indicated that MPH had reduced bitterness compared to SPH. These findings suggest that modifying soybean hydrolysis can improve protein digestibility, functional properties, and palatability, making it beneficial for nutraceutical applications (Jogi et al.).

Other methods, such as thermal treatment, can induce structural modifications in proteins, thereby altering their functional properties and enhancing their digestibility. The third article assessed the influence of thermal denaturation of whey protein isolates combined with chitosan for Pickering emulsion fabrication (Pu et al.). This study investigated how thermal denaturation of whey protein isolates (WPI) affects their properties when combined with chitosan (CS) to form Pickering emulsions. Three types of composite particles were prepared: WPI-CS (unmodified WPI with CS), DWPI-CS (thermally denatured WPI with CS), and D(WPI-CS) (WPI combined with CS followed by thermal denaturation). The composite particles formed larger aggregates with greater size and surface roughness compared to individual CS and WPI, indicating improved emulsification capacity. Emulsions stabilized with these composite particles had droplet sizes of 20.00 ± 0.15 μm (WPI-CS), 27.80 ± 0.35 μm (DWPI-CS), and 16.77 ± 0.51 μm [D(WPI-CS)]. Thermal stability tests showed that WPI-CS and DWPI-CS-stabilized emulsions had relatively better thermal stability than D(WPI-CS). Furthermore, the *in vitro* bioavailability of curcumin emulsion stabilized with WPI-CS was 61.18 ± 0.16%, significantly higher than the other two composite emulsions. This study provides valuable insights for designing customized WPI-based Pickering emulsions for use in the food and nutrition industries (Pu et al.).

Assessing amino acids and small peptides after protein modification is essential for understanding structural alterations and evaluating the efficiency of the applied method in improving digestibility. In the furth article, the UHPLC-QQQ-MS/MS method was utilized for the simultaneous quantification of 18 amino acids in various meat samples, providing valuable data that can be effectively applied to protein modification research. This study developed an innovative method using ultra-high-performance liquid chromatography coupled with triple quadrupole mass spectrometry (UHPLC-QQQ-MS/MS) to simultaneously quantify 18 amino acids in different meat types, including pork, pork feet, lean meat, fat, pork skin, beef, lamb, and chicken. The method proved to be fast, sensitive, and precise, with simplified sample preparation that avoids complex derivatization procedures. Results revealed that glutamate was the most abundant amino acid in all samples, reaching 20,300 μg/g in pork feet, while aspartate was the least abundant, with 0.0945 μg/g in pork and undetectable in some beef and lean meat samples. Multivariate analysis distinguished different meat types and components based on their amino acid profiles (Wang et al.). This method provides a valuable tool for evaluating the nutritional quality of meat products and has applications in protein chemistry, food science, and clinical medicine.

In summary, advancements in protein modification techniques, such as enzymatic hydrolysis and thermal treatment, play a crucial role in improving protein digestibility and functionality. The studies reviewed in this editorial demonstrate how novel proteases, dual hydrolysis, and thermal denaturation enhance protein bioavailability and sensory properties, particularly in plant- and dairy-based proteins. Additionally, the development of advanced analytical techniques, such as UHPLC-QQQ-MS/MS, provides valuable insights into amino acid composition and modification efficiency. These findings collectively contribute to optimizing protein utilization, supporting human nutrition, and promoting sustainable food solutions.
